# Experiences of Trauma and DNA Methylation Profiles among African American Mothers and Children

**DOI:** 10.3390/ijms23168951

**Published:** 2022-08-11

**Authors:** Veronica Barcelona, Yunfeng Huang, Billy A. Caceres, Kevin P. Newhall, Qin Hui, Jessica P. Cerdeña, Cindy A. Crusto, Yan V. Sun, Jacquelyn Y. Taylor

**Affiliations:** 1 Center for Research on People of Color, Columbia University School of Nursing, 560 West 168th St., New York, NY 10032, USA; 2 Biogen, 225 Binney Street, Cambridge, MA 02142, USA; 3 School of Medicine, Case Western Reserve University, 10900 Euclid Ave., Cleveland, OH 44106, USA; 4 Department of Epidemiology, Emory University Rollins School of Public Health, 1518 Clifton Road NE, Atlanta, GA 30322, USA; 5 MD-PhD Program, Yale School of Medicine, 367 Cedar St., New Haven, CT 06520, USA; 6 Department of Anthropology and Institute for Collaboration on Health, Intervention, and Policy (InCHIP), University of Connecticut, 354 Mansfield Rd., Storrs, CT 06269, USA; 7 Department of Psychiatry, Yale School of Medicine, 300 George St., New Haven, CT 06511, USA; 8 Department of Psychology, University of Pretoria, Private Bag x 20, Hatfield, Pretoria 0028, South Africa

**Keywords:** African Americans, DNA methylation, trauma, ACES, epigenomics, women

## Abstract

Potentially traumatic experiences have been associated with chronic diseases. Epigenetic mechanisms, including DNA methylation (DNAm), have been proposed as an explanation for this association. We examined the association of experiences of trauma with epigenome-wide DNAm among African American mothers (*n* = 236) and their children aged 3–5 years (*n* = 232; N = 500), using the Life Events Checklist-5 (LEC) and Traumatic Events Screening Inventory—Parent Report Revised (TESI-PRR). We identified no DNAm sites significantly associated with potentially traumatic experience scores in mothers. One CpG site on the *ENOX1* gene was methylome-wide-significant in children (FDR-corrected q-value = 0.05) from the TESI-PRR. This protein-coding gene is associated with mental illness, including unipolar depression, bipolar, and schizophrenia. Future research should further examine the associations between childhood trauma, DNAm, and health outcomes among this understudied and high-risk group. Findings from such longitudinal research may inform clinical and translational approaches to prevent adverse health outcomes associated with epigenetic changes.

## 1. Introduction

Potentially traumatic experiences over the life course are common and have a strong, cumulative impact on adult health [1]. These traumatic experiences may occur during childhood—commonly referred to as Adverse Childhood Experiences (ACEs)—or later in development. Over half of Americans endure at least one traumatic experience early in life and African Americans and Latinos are more likely to sustain multiple childhood traumas [2,3]. Each additional exposure to childhood trauma increases a person’s likelihood of developing a life-threatening health condition in adulthood, such as substance abuse, suicidality, cancer, liver disease, and ischemic heart disease [4,5,6,7]. This powerful, graded relationship between childhood trauma and adverse health outcomes in adulthood suggests that psychosocial stress modulates physiologic systems during development to shape health trajectories.

Early life adversity is an important social determinant of health that has been linked to the disruption of stress-sensitive systems, leading to disease in adulthood [8,9]. These include neuroendocrine, inflammatory, and metabolic dysregulation, which have been implicated in the development of cardiovascular disease (CVD) [10]. In the National Survey of Child Health, consisting of over 84,000 children, African Americans were exposed to more adversities than White children [11]. Research suggests that ACEs may be associated with lower socioeconomic status [12] and that urban African Americans are especially vulnerable to ACEs [5,13]. African Americans who experience ACEs have higher rates of obesity, inflammation, depressive symptoms, and substance use [5,13,14].

A growing body of research indicates that potentially traumatic experiences in adulthood, such as intimate-partner violence, are associated with negative health outcomes in women. Analyses of population-based data have found that intimate-partner violence, specifically emotional abuse, is associated with a 24% increased rate of incident hypertension in women [15]. Similarly, analyzing data on over 60,000 women enrolled in the Nurses’ Health Study II, Mason and colleagues found that psychological intimate-partner violence was associated with a 61% increased incidence of type 2 diabetes [16]. Further, a retrospective cohort study conducted in the United Kingdom found that women exposed to intimate-partner violence had an increased risk of cardiovascular disease events and all-cause mortality [17]. Thus, both ACEs and adulthood traumas bear upon health and development, which is particularly concerning for vulnerable populations, such as African Americans.

Epigenetic mechanisms have been proposed as a proximate means by which environmental factors, such as psychosocial stress, may influence health [14,18,19,20]. In particular, DNA methylation (DNAm), or the addition of a methyl group to a cytosine residue, may modify the binding of transcription factors to regulate gene expression levels [21]. In response to stress, including potentially traumatic experiences, epigenetic modifications may alter the function of molecular pathways to induce lasting health consequences. Epigenetic processes may embed environmental experiences into the genome through enzymatically catalyzed modifications of DNA [22]. Growing evidence suggests that childhood adversity is associated with persistent changes in DNAm [23,24,25]; however, to date, fewer studies have investigated the relationship between potentially traumatic experiences across the life course and alterations throughout the methylome [26,27,28]. Epigenome-wide association studies (EWAS) allow systematic analysis of associations between potentially traumatic experiences and variations in DNAm, which may help identify targets of epigenetic regulation in response to psychosocial stress during development.

In this study, we assessed the relationship between potentially traumatic experiences and methylome-wide DNAm in African American women and their children. Given that little is known about the role of DNAm in mediating the health effects of potentially traumatic experiences in African Americans, we seek to fill a critical gap in the current understanding of trauma-associated health disparities in this population.

## 2. Results

In the total study sample of 250 mothers, 4 were missing data for the LEC total score and 3 were missing maternal smoking information. In addition, one child in our sample was excluded due to missing age. We also excluded participants with missing data after QC/normalization, leaving a total of 236 mothers and 232 children in the EWAS analysis of LEC. Characteristics of the sample are summarized in Table 1. The mean age of our study sample was 31.2 years for mothers and 3.7 years for children. Most mothers (77.9%) indicated that they were not current cigarette smokers. The majority of mothers (57.7%) had completed some college or higher levels of education and reported an annual income of less than or equal to $15,000 (46.4%). Mothers reported having Medicaid most commonly (62.9%) and 22 identified as Hispanic/Latina (9.3%). Marital status was most frequently indicated as single (65.5%) and more than half of the children (58.9%) were females. Mothers reported experiencing at least one traumatic event in their own (70.2%) and their children’s (49.1%) lifetimes. The mean value for lifetime potentially traumatic experiences for mothers was 2.0 (range 0–9) and for children, 0.9 (range 0–7).

**Figure 1:**
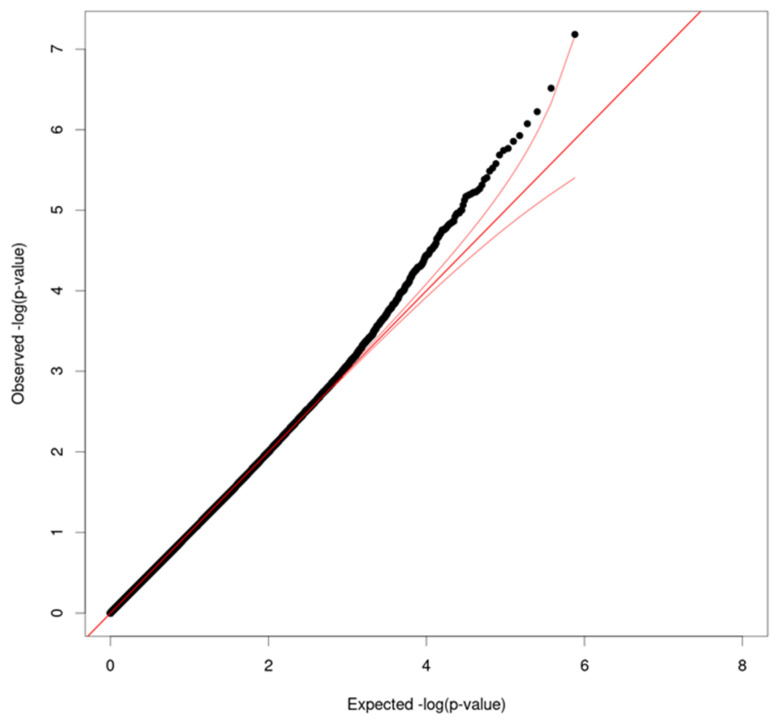
Q-Q plot of epigenome-wide associations with Traumatic Events Screening Inventory—Parent Report Revised (TESI-PRR) for children. Associations of DNAm level at each CpG site with TESI-PRR were tested using linear mixed model adjusted for age, child sex, smoking, cell-type proportions, and top-ten principal components. The total sample size is *n* = 232. Observed −log10(p) (*y*-axis) was plotted against expected −log10(p) derived from a uniform distribution (*x*-axis). Overall, the distribution showed a well-controlled type I error rate. Red straight line is *y* = *x* and the red curved lines indicate the 95% confidence interval for the expected −log10(p).

**Figure 2:**
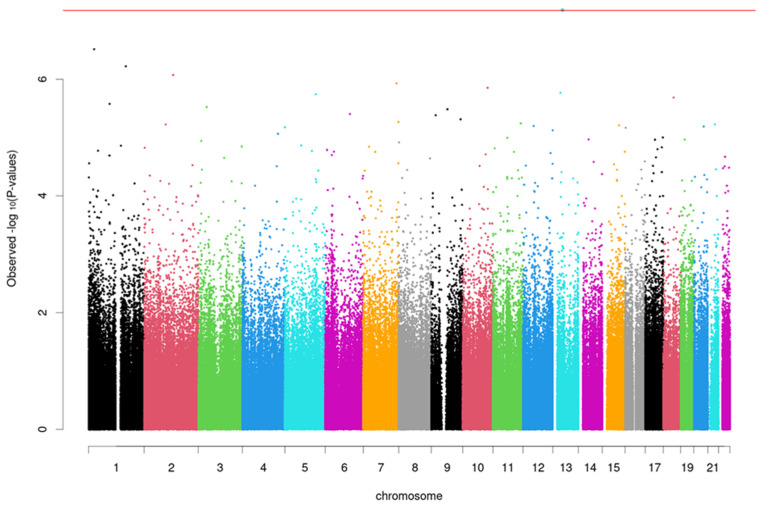
Manhattan plot of epigenome-wide associations with Traumatic Events Screening Inventory—Parent Report Revised (TESI-PRR) for children. Associations of DNAm level at each CpG site with TESI-PRR was tested using linear mixed model adjusted for age, child sex, smoking, cell-type proportions, and top-ten principal components. The total sample size is *n* = 232. −log10(p) of each CpG site is plotted against its genomic position (hg19). Red line indicates the *p*-value cutoff for epigenome-wide significance (print in color).

In EWAS analyses, no CpG sites were significantly associated with the LEC for mothers or children. Additional EWAS for TESI-PRR revealed one epigenome-wide significant site after FDR correction for children (*p* = 0.055), on the ENOX1 gene, cg10448831 probe (Chr 13, basepair position 43930401, beta = −0.0085, SE = 0.0014). The Q-Q and Manhattan plots of the epigenome-wide associations for children are presented in Figure 1 and Figure 2. Top CpG sites from each of the analyses conducted are presented in Supplementary Tables S1–S4.

## 3. Discussion

In this EWAS study, we examined the impact of lifetime and childhood potentially traumatic experiences in African American mothers and their young children. We found no sites that were statistically significant for the LEC among mothers or children. However, we did find one CpG site on the ENOX1 gene (cg10448831), which was significantly associated with trauma in children in this sample. This is a protein-coding gene, which is involved in electron transport in plasma membranes [29]. Phenotypically, ENOX1 has been associated with mental illnesses, such as schizophrenia, unipolar depression, and bipolar disease. Prior studies documented a significant association between this gene, child trauma, and mental illness [30,31].

This finding is significant as it demonstrates DNAm changes in very young children after parent-reported traumatic experiences. A recent systematic review identified an association between lower childhood socioeconomic status and increased risk for ACEs, indicating the importance of addressing socioeconomic context in policy efforts to mitigate childhood trauma [12]. In addition, previous research in mothers and children under age 3 years has reported that mothers with three or more ACEs had children with higher levels of anxiety, aggression, hyperactivity, and negative affect [32]. In that study, authors suggest that improved parenting support for individuals experiencing traumatic experiences may reduce the risk of poor outcomes in children resulting from poor postpartum mental health, lower confidence as a parent, and maladaptive coping [32]. Indeed, several studies have observed a significant association between ACEs and parenting stress [33,34,35,36] and we documented the link between parenting stress and DNAm in previous analyses in this sample [37,38]. Further research attending to both the biological and socioecological mechanisms of intergenerational effects on methylation can elucidate these relationships.

It is also well documented that childhood trauma is associated with psychosis later in life [39]. Though similar rates of mental illness, such as major depressive disorder, have been observed among African Americans as their counterparts of other races and ethnicities [40], some research indicates that the prevalence may be higher and may be explained by underdiagnosis and lack of access [41]. This warrants continued investigation to elucidate mitigating factors and to identify interventions and strategies to reduce disparities.

In addition, genetic risk factors for illness do not fully explain disease expression and, in fact, may be overridden by environmental exposures. Other epigenome-wide studies demonstrated associations between DNAm and psychiatric illnesses [42], highlighting the important role of psychological stressors (such as trauma) in altering the epigenome. Trauma may affect immune factors, such as cytokine regulation, on the pathway to DNAm [43]. Little is known about the pathways involved and how psychological and environmental exposures influence DNAm, especially among African Americans. Additional social exposures, such as racism, should be investigated as well in future studies, to examine the possible interactive or potentiating effects with trauma on DNAm and the expression of disease. Further, it will be important to investigate the protective factors that may be involved in resiliency and how coping mechanisms may be associated with DNAm and disease prevention.

Strengths of the current study include maternal and child DNAm measurement, an all African American sample, and examination of effects of traumatic experiences on mothers and young preschool-aged children, not typically represented in these studies. There may be limited generalizability of findings as this was a community sample of African Americans who were relatively homogenous in socioeconomic status. In addition, mothers may have underreported traumatic experiences due to social desirability bias or due to the sensitive nature of the questions; however, if this was the case, it would have biased our associations in the direction of the null.

Saliva was the tissue of choice for the epigenomic analyses conducted in this study due to the ease of collection and avoidance of invasive procedures. This is especially important as our study population included young children. Previous work examining the consistency of DNAm measurement in different tissues using adult African Americans suggested that saliva may be more heterogeneous than blood, yet there was a positive correlation overall [44]. These findings may, consequently, support our tissue choice of saliva, as ACEs are associated with negative mental health outcomes [45]. Another study comparing saliva to white blood cells isolated from blood samples from young girls showed that saliva typically displayed lower DNAm levels than in white blood cell DNA [46]. This may suggest that our data are more conservative and, thus, could even be lacking some additional CpG sites.

In conclusion, we found that childhood traumatic experiences were associated with DNAm changes among African American children at one significant CpG site. Future research should further examine the associations between childhood trauma, DNAm, and health outcomes among this understudied and high-risk group. Findings from such longitudinal research may inform clinical and translational approaches to preventing adverse health outcomes associated with epigenetic changes.

## 4. Materials and Methods

### 4.1. Study Sample

Data for this study were collected from the Intergenerational Impact of Genetic and Psychological Factors on Blood Pressure Study (InterGEN), which examined genetic, epigenetic, and environmental factors associated with high blood pressure among Black/African American mothers and their young children (N = 500). Extensive details on recruitment are described elsewhere [47]. In brief, mothers who had a child between the ages of 3 and 5 years and self-identified as African American and/or Black were recruited from early care and education centers in Connecticut from 2014 to 2019. Mother–child dyads were followed for 2 years and four interviews were conducted at six-month intervals for data collection. All study procedures and measures were approved by the Institutional Review Boards at Yale University (IRB# 1311012986) and Columbia University (IRB# AAAS9653). The datasets for the current study are available from the corresponding author on reasonable request. All methods were carried out in accordance with relevant guidelines and regulations.

### 4.2. Survey Measures

At the baseline interview, mothers completed background demographic information, the Life Events Checklist (LEC) [48] and the Traumatic Events Screening Inventory—Parent Report Revised (TESI-PRR) [49]. The LEC is an adult trauma measure designed to screen for potentially traumatic events over the respondent’s lifetime. The instrument assesses exposure to 16 events known to potentially result in distress and one additional question assessing any other experience not included in the questionnaire. The TESI-PRR is 24-item measure of childhood trauma exposure that is completed by a parent/caregiver. Both surveys cover potentially traumatic events such as current and previous injuries, hospitalizations, domestic violence, disasters, accidents, and physical and sexual abuse. Scores range from 0 to 17 for the LEC and 0 to 24 for the TESI-PRR, with higher scores indicating exposure to more potentially traumatic events. Prior research demonstrated excellent internal consistency for the LEC alpha = 0.94; reliability is not yet available for the TESI-PRR [50,51]. All survey measures were administered via Audio Computer-Assisted Self-Interview (ACASI) software.

### 4.3. Potential Confounders

Mothers self-reported age in years, whether they were current cigarette smokers (yes/no), and other demographic data were assessed at the initial interview. Child age was calculated from date of birth and date of interview. We controlled for maternal and child age and maternal smoking, which are accepted confounders in epigenetic studies. We also adjusted for batch effects and potential heterogeneity in cell proportions from saliva using the reference-free EWAS (Epigenome-Wide Association Studies) method [52].

### 4.4. DNA and Epigenetic Processing

Saliva was collected from participants for DNA analysis using the Oragene (OG)-500 format tubes [53]. This procedure requires that mothers and children spit into the tube until they reach the fill line (2 mL). DNA extraction and analysis were carried out per standard operating procedure guidelines using ReliaPrep kits; additional details on DNA processing have been published elsewhere [54]. To protect participant confidentiality and ensure accuracy of analyses, tubes and plates containing DNA were barcoded and entered into a computerized laboratory freezer inventory. Barcode scanning was also used for DNA pipetting with robotic workstations to track the transfer of the biological materials from tubes to plates. This method ensures accurate identification of each participant’s DNA for correct merging to genotype calls.

We quantified DNAm using the Illumina Infinium Methylation EPIC (850K) BeadChip and then performed quantile normalization of beta values for autosomal CpG sites. We applied laboratory-based quality control procedures (missing rate < 10% and no sex mismatch) to each individual sample. We excluded CpG sites if they had a missing rate greater than 10% (*n* = 3343), overlapped with single-nucleotide polymorphisms (*n* = 87,074), or were listed in the recent Illumina quality notice (*n* = 977). In total, 756,448 autosomal sites were included in the association analyses.

### 4.5. Statistical Analyses

EWAS analyses of potentially traumatic experiences (LEC and TESI-PRR) were conducted among mothers and children separately. DNAm beta-values were modeled as the dependent variable. Linear mixed-effects models with two random effects (batch and chip) were applied to explore the epigenetic associations with LEC and TESI scores in mothers and children, adjusted for age and maternal smoking. Sex was also adjusted in the analyses among children. False discovery rate (FDR) was used to correct for multiple comparisons and a cutoff of FDR < 0.05 was used to declare statistical significance. We conducted principal component analysis (PCA) using 164,613 CpGs within 50 bp of SNPs to correct for potential population stratification. The top-ten PCs were included as covariates in the model [55]. Cell-type proportions estimated using the “RefFreeEWAS” method were included as covariates [51]. All analyses were conducted in the R statistical computing environment [56]. DNAm processing and QC was conducted using Bioconductor package “minfi” (version 1.36,0). Linear mixed models were run using R package “nlme” (version 3.1-141). Cell-type proportions were estimated using R package “RefFreeEWAS” (version 2.2) and PCA was conducted using R function “prcomp” in the “stats” package (version 3.6.1).

## Figures and Tables

**Table 1:** Baseline characteristics of mothers and children (*n* = 236 mothers, *n* = 232 children).

	*n* * (Mean)	% (S.D.)
Maternal characteristics (*n* = 236)		
Age (mean, S.D.)	31.2	5.6
20–29	99	41.9
30–39	118	50.0
40–49	19	8.0
Current smoker No	183	77.9
Yes	52	22.1
Education <High School	13	5.5
High School graduate	86	36.6
Some college	78	33.1
Associates/College Grad or higher	58	24.6
Annual household income ≤$15,000	106	46.4
>$15,000–$50,000	100	43.8
>$50,000	22	9.6
Health insurance type Private	32	13.6
Medicaid	148	62.9
Government/ACA	35	14.8
None	13	5.5
Hispanic/Latina ethnicity No	213	90.2
Yes	22	9.3
Marital Status		
Married	56	23.8
Single	154	65.5
Divorced/Separated	13	5.4
Living with a partner	12	5.1
LEC scores (Total lifetime trauma events) (mean, S.D.)	2.0	2.1
None reported	66	29.7
At least one event	156	70.2
Child characteristics (*n* = 232)		
Sex Male	92	39.7
Female	140	60.3
Age (years), mean (S.D.)	3.7	0.7
TESI scores (Total child trauma events), mean (S.D.)	0.9	1.4
None reported	117	50.9
One or more	113	49.1

* Numbers may not sum to 100% due to missing data, S.D. = standard deviation.

## Data Availability

Data are available from authors upon reasonable request.

## Supplementary Materials

The following supporting information can be downloaded at: https://www.mdpi.com/article/10.3390/ijms23168951/s1.
